# Nanomedicine as an Emerging Technology to Foster Application of Essential Oils to Fight Cancer

**DOI:** 10.3390/ph15070793

**Published:** 2022-06-25

**Authors:** Khaled AbouAitah, Witold Lojkowski

**Affiliations:** 1 Medicinal and Aromatic Plants Research Department, Pharmaceutical and Drug Industries Research Institute, National Research Centre (NRC), 33 El-Behouth St., Dokki, Giza 12622, Egypt; 2 Laboratory of Nanostructures and Nanomedicine, Institute of High Pressure Physics, Polish Academy of Sciences, Sokolowska 29/37, 01-142 Warsaw, Poland

**Keywords:** cancer, delivery system, essential oil, loading and controlled release, nanoformulation, nanomedicine

## Abstract

Natural prodrugs extracted from plants are increasingly used in many sectors, including the pharmaceutical, cosmetic, and food industries. Among these prodrugs, essential oils (EOs) are of particular importance. These biologically active volatile oily liquids are produced by medicinal and aromatic plants and characterized by a distinctive odor. EOs possess high anticancer, antibacterial, antiviral, and antioxidant potential but often are associated with low stability; high volatility; and a high risk of deterioration with exposure to heat, humidity, light, or oxygen. Furthermore, their bioavailability is limited because they are not soluble in water, and enhancements are needed to increase their potential to target specific cells or tissues, as well as for controlled release. Nanomedicine, the application of nanotechnology in medicine, may offer efficient solutions to these problems. The technology is based on creating nanostructures in which the natural prodrug is connected to or encapsulated in nanoparticles or submicron-sized capsules that ensure their solubility in water and their targeting properties, as well as controlled delivery. The potential of EOs as anticancer prodrugs is considerable but not fully exploited. This review focusses on the recent progress towards the practical application of EOs in cancer therapy based on nanotechnology applications.

## 1. Introduction to Essential Oils

Attention to natural agents (also known as natural products or natural prodrugs) as modern medical therapeutics is increasing, with the aim of using them as replacements for synthetic drugs. In particular, natural active agents derived from plant sources have a long history, and in addition to new ones being sought, known candidates are being repurposed for potential applications in many diseases and entered into clinical trials [1]. Essential oils (EOs) are defined by the European Pharmacopoeia 7th edition as odorant products characterized by a complex composition obtained from a botanically defined plant raw material and derived by steam, dry distillation, or a mechanical method without any heating [2]. EOs [3] are mainly composed of terpenes [4,5]. An essential oil contains several compounds that contribute to its therapeutic value, some major and some minor, and that are not derived from a specific common chemical structure, as with other natural agents, such as flavonoids and alkaloids. From the chemical composition perspective, EOs are complex mixtures originally constituted by mono- and sesquiterpene hydrocarbons with their oxygenated derivatives; in addition, they have aliphatic aldehyde, alcohol, and ester structures [6]. Produced by plants as part of their secondary metabolism, EOs can be obtained from hundreds of herbs and plants and are well-known for their use in traditional medicine. Among the many EO sources are sage, lavender, clove, eucalyptus, anise, black seed, cumin, cinnamon, citrus, cardamom, ginger, rosemary, geranium, onion, garlic, lemon, and peppermint. Medicinal and aromatic plants containing EOs, such as peppermint, thyme, and sage, have been industrially cultivated to provide a sustainable source of these oils. 

### 1.1. Methods of EOs Obtaining

EOs can be obtained from plant material through several extraction methods, including hydro-distillation, steam distillation, cold pressing, solvent extraction, ultrasound, and microwave-assisted processes [7,8,9,10,11]. A special feature of EOs is their diversity of chemical structures and sources. An estimated 17,000-plus plant species containing EOs occur worldwide [12]. Of the more than 3000 EOs that have been identified, only 10% are commercially produced. The global market for EOs is predicted to reach USD 13.94 billion by 2024 [13]. EOs are assumed to have diverse therapeutic actions and medical applications because of their biocidal activities (bactericidal, virucidal, and fungicidal) and potential as anticancer, cardiovascular, antioxidant, analgesic, and antidiabetic agents [14,15].

**Figure 1:**
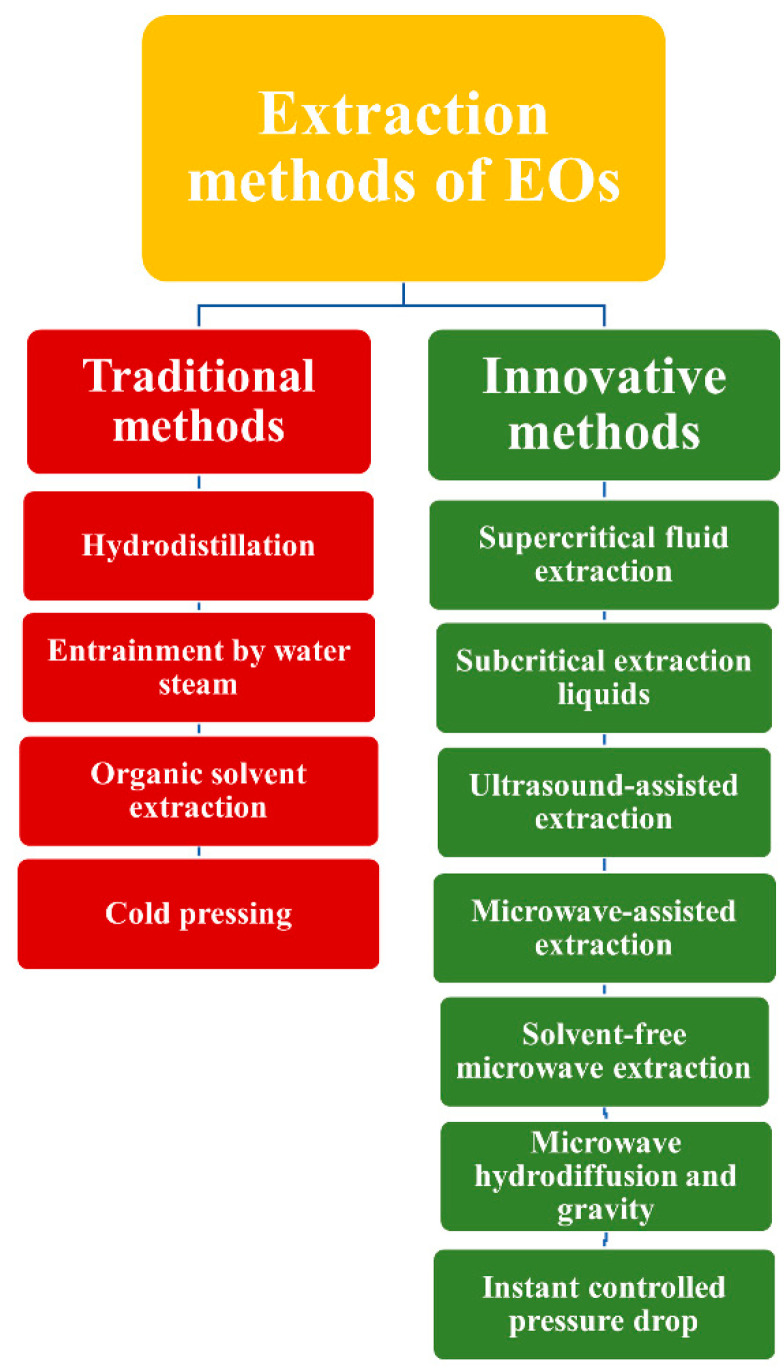
Possible extraction methods for EOs classified into traditional and innovative approaches [2].

The methods to extract EOs from plant materials can be classified as traditional or innovative, as shown in Figure 1 [2]. The time needed for extraction, EO yield, energy consumption, and quality can vary with the extraction method, as has been thoroughly covered previously [7,10,16,17,18,19,20,21,22,23,24,25,26,27]. 

### 1.2. Applications of EOs in Health Care

With a long history in many cultures, EOs represent an important domain within traditional medicines worldwide, used for different purposes depending on each culture [28]. As an example, the ancient Egyptians used EOs as early as 4500 BCE for cosmetics and ointments [6]; they developed a formulation of herbal mixtures of, e.g., aniseed, cedar, onion, myrrh, and grapes for perfume or medicinal use. By the middle of the 20th century, EOs were somewhat limited when using in nonmedical areas [29], although extensive investigations have been done on their pharmacological and biological activities [30]. 

Their utility has been explored in the pharmaceutical, agriculture, and food industries; for sanitary purposes; and for use in cosmetics and perfumes [31]. With regard to biomedical applications, they are considered for various formulations, making them an important source for innovative strategies against, e.g., microbial infections [31]. Their use is also increasing in the pharmaceutical sector, with EO preparations being developed in various dosage forms, including capsules, syrups, ointments, creams, suppositories, aerosols, and sprays [2]. 

Directly applied EOs may play an important role as additional treatments supplementing cancer treatment. These additional treatments include antibacterial, antifungal, antiviral, anti-inflammatory, anti-lice, and antioxidant effects [32]. One of the most widely explored applications of EOs is aromatherapy because of its potential curative effects [23,32,33]. The term aromatherapy was introduced in 1936 by the French chemist Gattefossé [34]. It can be classified as cosmetic aromatherapy, message aromatherapy, medical aromatherapy, olfactory aromatherapy, and psycho-aromatherapy [35]. Therefore, it can be beneficial to manage pain, nausea, vomiting, anxiety, depression, stress, insomnia, respiratory, dementia, and others [36]. These functions are important to supplement the conventional anticancer clinical methods. EO inhalation and oral administration are tested in preclinical and clinical trials [37] to treat anxiolytic states. Additionally, in respiratory diseases, pain management, and stress alleviation, gentle massage, inhalation, or the oral intake of capsules can be applied [38]. Boukhatem et al. [39] showed promising results during in vivo tests of lemon grass EO applications for curing fungal infections and skin inflammation. Similarly, in a preclinical study, cumin EO showed promising effects when applied perorally in human campylobacteriosis, a food-borne infection [40]. One of most common side effects during chemotherapy is nausea and vomiting. It was found that the inhalation of ginger EO could be used as a complementary treatment for cancer patients [41,42,43]. In breast cancer, aromatherapy massage revels an anxiolytic effect and ameliorates the immunologic state in breast cancer patients [44]. Additionally, massage aromatherapy was found to be useful for relieving neuropathic pain and fatigue in cancer patients [45]. 

### 1.3. Methods of EOs Administration

**Figure 2:**
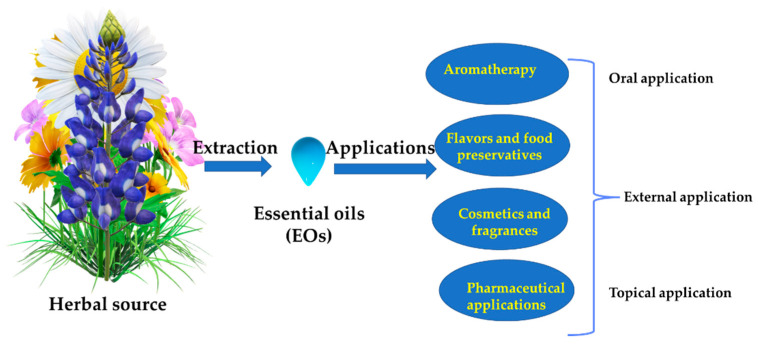
Outline of various applications and administration routes for EOs.

EOs can be administered through various routes, including external, oral, and topical, as shown in preclinical and clinical applications (Figure 2) [37,38,39,46]. The routes of EO administration were described in detail by Vostinaru et al. [35]. Schilcher [47] divided the observed effects of EOs depending on the route of administration. Hyperemic, anti-inflammatory, antiseptic, granulation stimulating, deodorizing, and other effects are observed when EOs are applied externally. Expectorating, appetite stimulatory, choleretic, anti-inflammatory, antiseptic, sedative, and disinfectant effects are observed when EOs are applied orally. 

An interesting approach is to develop codelivery systems by combining EOs with clinical anticancer drugs to reduce the side effects and enhance the solubility of anticancer drugs [48], including unusual EO substances [49].

The encapsulation of EOs is the technology of choice for enhancing their medical activity [50]. This strategy can lead to their extended and controlled release over a longer time [6] while enhancing their therapeutic efficiency [51]. The goal is to formulate delivery systems that allow for controlled release, improve physical stability, protect against off-target activity, reduce volatility, increase therapeutic activity, reduce toxicity, and further improve patient adherence and convenience [52,53,54]. Additionally, a pharmaceutical preparation with a sustained–release pattern can extend the plasma concentration within the therapeutic treatment window, effectively increasing the efficacy [55]. Encapsulated EOs can be released through several mechanisms, including mechanical action, heat changes, diffusion from nano-/microparticles, and stimuli-responsive release, by triggers such as pH, the biodegradation of nanocarriers, and dissolution [53]. Generally, these strategies can be classified based on the features related to the fabrication methods or materials, among others. As far as the method of encapsulation is concerned, spray drying, coacervation, nano-/microemulsions, nanoprecipitation, high pressure, homogenization, coatings, freeze-drying, in situ polymerization, supercritical fluid, and others have been used [56,57,58]. Encapsulation can also be categorized based on chemical, physicochemical, and mechanical methods [59,60,61,62,63]. 

### 1.4. EO Side Effects

Many EOs are classified as Generally Regarded as Safe (GRAS) by the U.S. Food and Drug Administration (FDA) and are included in the Everything Added to Food in the US list [13]. 

However, the misuse of EOs may lead to side effects such as allergies, intoxication phototoxicity, photosensitivity, necrotic, narcotizing, abortion-provoking, nephrotoxic, hepatotoxic, carcinogenic, and other effects [35,47]. There is still insufficient knowledge on the safety profiles of EOs [35,36]. 

### 1.5. Anticancer Activity of EOs

**Figure 3:**
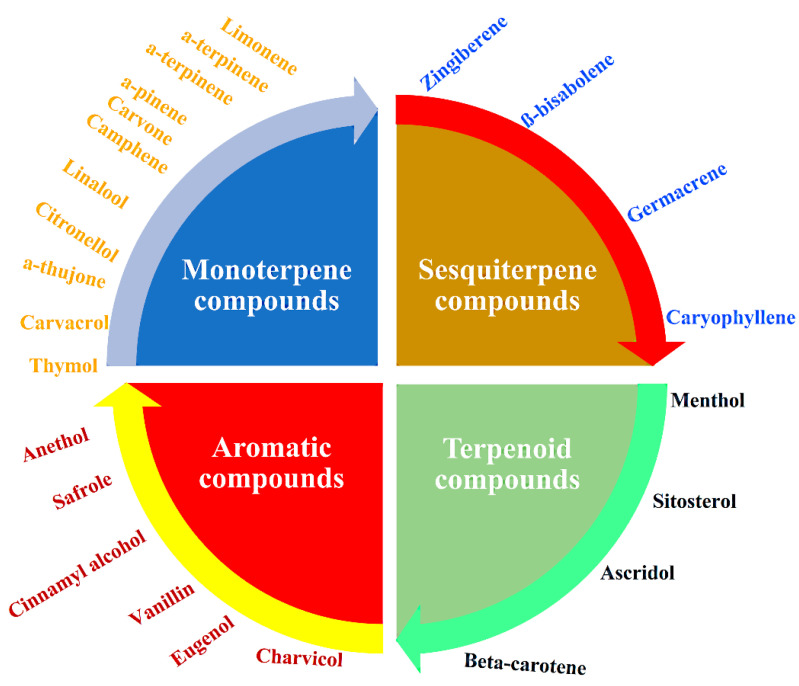
The main classes of EOs (monoterpenes, sesquiterpenes, terpenoids, and aromatic compounds), with some examples of their compounds [65,66].

The bioactivity potential of EOs is directly related to the quality and quantity of their chemical constituents [64]. Multiple anticancer effects are possible because of the diversity of the chemical constituents of EOs, including monoterpenes, oxygenated monoterpenes, sesquiterpenes, oxygenated sesquiterpenes, diterpenes, and phenolics, especially phenylpropanoids (C_6_–C_3_). Figure 3 shows the main types of EO compounds, with some examples. The anticancer potential has been explored for EOs and for their constituent compounds, such as carvacrol, linalool, and thymol [65,66].

**Figure 4:**
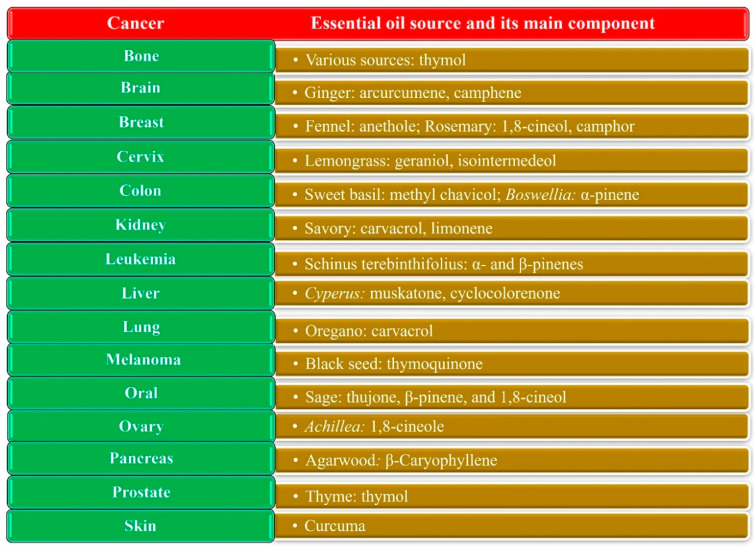
Examples of EOs with anticancer effects in cancers of the brain [74], colon [75,76], breast [77,78], skin (melanoma) [79,80], blood (leukemia) [81,82], oral tissues [83,84], bone [85], cervix [86], lung [87], prostate [88], kidney [89], liver [90], ovary [91], and pancreas [92].

Figure 4 gives some examples of EOs and their components, with applications in various cancers. Several excellent reviews have been published on this topic [65,67,68]. The literature indicates that EOs operate by various mechanisms; however, the main one is apoptosis via different signaling pathways [66,69,70]. The other mechanisms include cell cycle arrest, antimetastatic and antiangiogenic activity, the induction of reactive oxygen and nitrogen species, DNA repair modulation, antiproliferative activity, tumor suppressor proteins, transcription factors, and enzymes [71]. For instance, Bayala et al. [65] reported that the anticancer activities of EOs could arise from their effects on reactive oxygen species (ROS) because of the relationship between oxidation and inflammation leading to cancer initiation and progression [72,73]. 

### 1.6. Limitations of Present Technology

**Figure 5:**
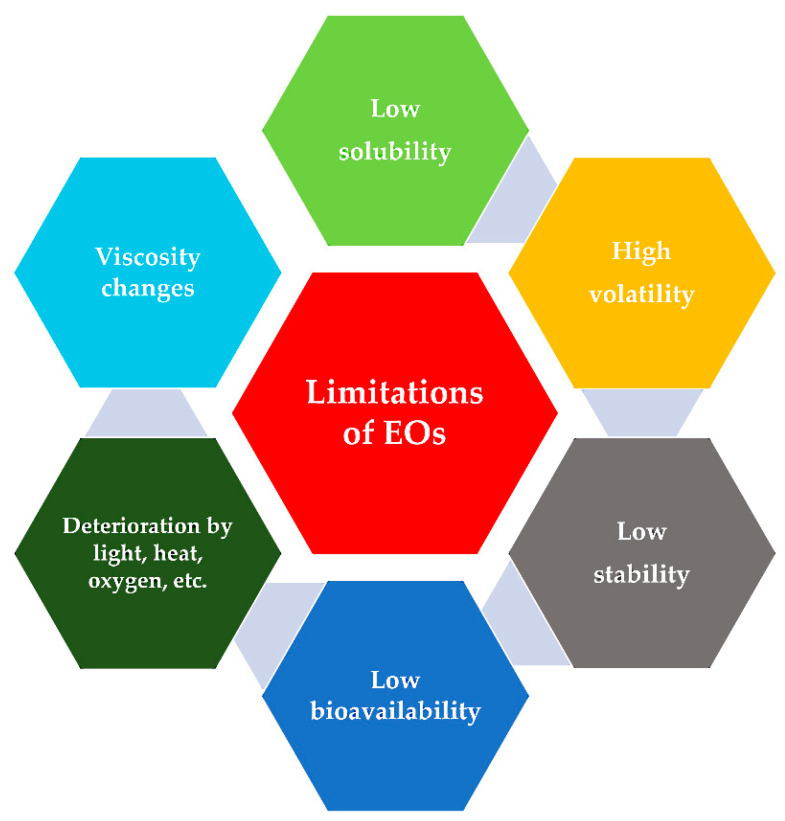
Schematic diagram highlighting the major limitations of Eos that prevent their use in clinical applications.

To date, the anticancer or antibacterial properties of hundreds of EOs remain only partially or not at all realized because of limitations preventing the exploitation of their possible applications and advantages. The limitations include low stability; high volatility; and a high risk of deterioration on exposure to direct heat, humidity, light, or oxygen [93]. Other properties that need mitigation for use in EOs include low water solubility (because of their hydrophobic nature) and low bioavailability (Figure 5). 

One of the strategic research directions of nanomedicine is to improve targeted and/or controlled drug delivery [49,56,94,95,96,97,98,99,100,101,102]. In addition, there is a need to improve EO stability and bioavailability along with cancer targeting through delivery system [103].

## 2. Nanomedicine Application to Enhance EOs Anticancer Efficiency

To our knowledge, this review is the first to totally focus on the application of nanotechnology to enhance the medical potential of EOs in anticancer applications. Our goal is to provide an information base and highlight the main research directions in the application of nanotechnology to enhance the anticancer effects of EOs. In this overview, we emphasize EO loading and controlled-release strategies; delivery systems based on nanocarriers (e.g., polymeric, lipids, and inorganic nanostructures); and anticancer delivery designs in vitro and in vivo, closing with challenges and future perspectives. 

### Nanodelivery Systems for EOs

In the last few years, a variety of nanodelivery systems have been designed to renew and expand the use of EOs. For example, Ali et al. [101] used nanosuspensions and nanocapsules of *Origanum glandulosum* EO constituents with sodium alginate. They showed that the nanocapsules exhibited a stronger anticancer effect against the liver cancer cell line HepG2 than either free EO or a nanoemulsion. Sousa et al. [104] illustrated that the encapsulation of EO compounds in silica modulated volatile compound releases. Additionally, using nanoemulsification to target drug-resistant cancers was explored [105]. 

**Figure 6:**
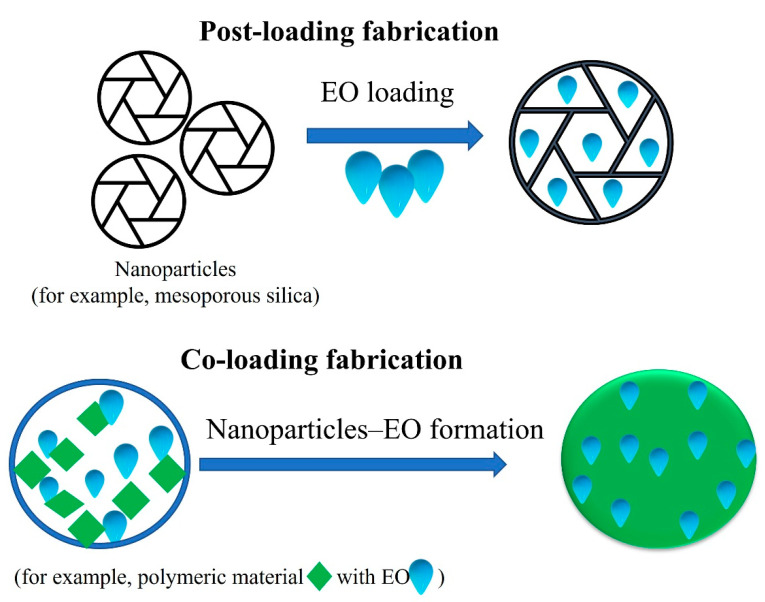
Schematic representation of two loading strategies that can be efficiently used for EO nanoformulations: post-loading fabrication and co-loading fabrication. The first approach is more likely for inorganic materials, and second approach is more likely for organic materials.

**Figure 7:**
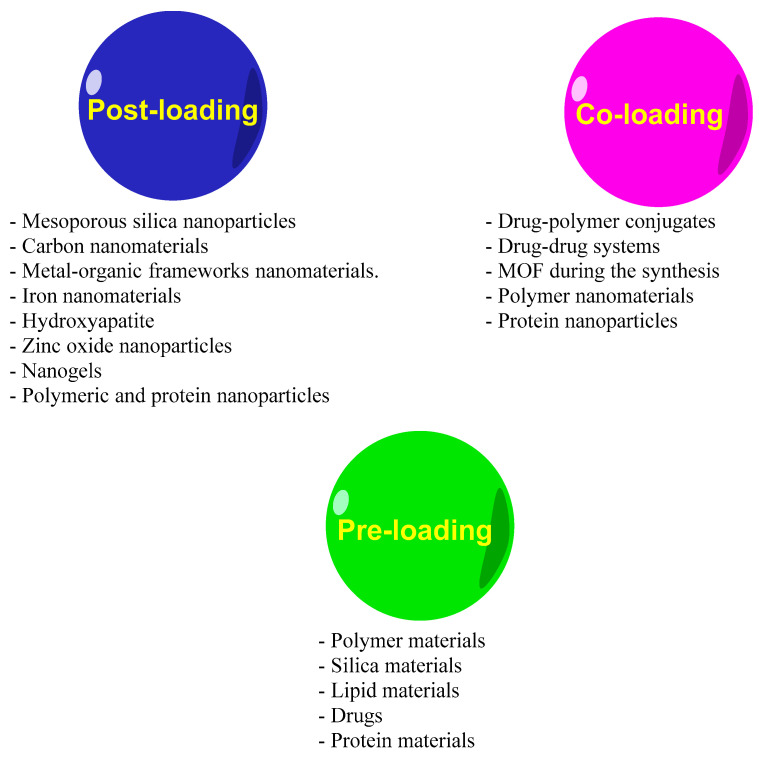
Materials used in the three drug-loading strategies based on Liu et al. [106].

In many cases, the original drugs must be modified to be loaded into nanocarriers prior to administration [55]. Several reviews have classified the loading strategies of drugs and therapeutic agents in different ways, based on the variety of carrier types and drug features. In one classification, Liu et al. [106] divided nanoparticle drug-loading strategies into three categories: post-loading, co-loading, and pre-loading. Post-loading references first preparing the nanoparticles, then loading them; co-loading refers to loading the drug during fabrication of the nanoparticles; and pre-loading refers to producing drug nanoparticles at the first stage and fabricating a shell that stabilizes and protects them in the second stage [106]. For EO loading, we believe that post-loading fabrication (initially, fabricate nanoparticles and then use EO loading to obtain the formulation) and direct loading fabrication (nanoparticle materials and EO directly used for the formulation) can be the first-choice approach for loading (Figure 6). The nanocarrier materials to be used based on these three strategies are shown in Figure 7 [106]. We believe that, for inorganic nanocarriers, the post-loading fabrication and, for polymeric nanocarriers, the co-loading fabrication approaches are optimal. Wang et al. [55] discussed a nanocarrier classification according to the loading approaches, i.e., molecular-level loading, surface loading, matrix loading, and cavity loading. These strategies are also tenable for EO delivery formulations.

**Figure 8:**
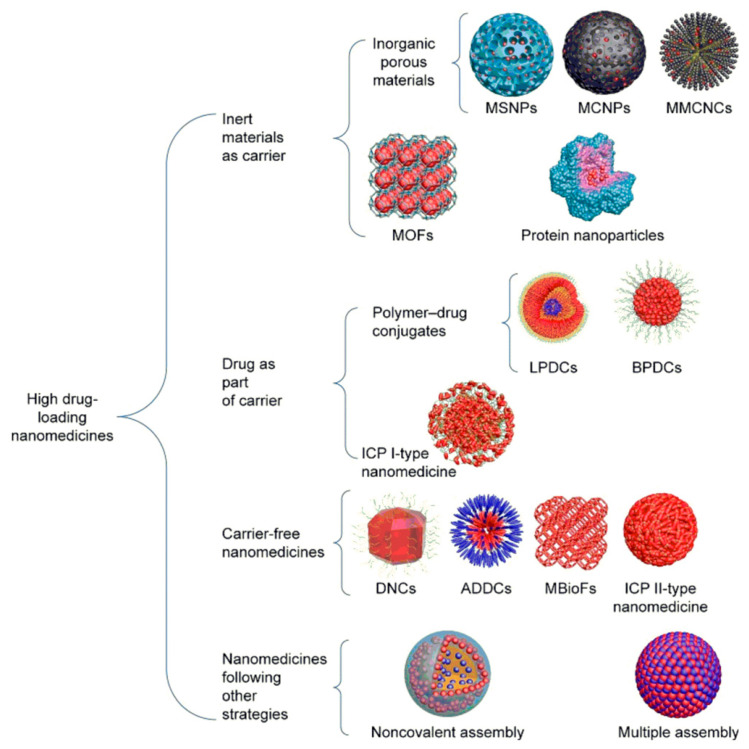
Schematic representation of the fabrication strategies of nanomedicines with a high drug-loading capacity. MSNs (mesoporous silica nanoparticles), MCNPs (mesoporous carbon nanoparticles), MMCNCs (mesoporous magnetic colloidal nanocrystal clusters), MOFs (metal organic frameworks), LPDCs (linear (polymer–drug conjugates)), BPDCs (branched PDCs), ICPs (infinite coordination polymers), DNCs (drug nanocrystals), ADDCs (amphiphilic drug–drug conjugates), and MBioFs (metal–biomolecule frameworks). Reprinted from Ref. [107]. Copyright 2017 Shen et al. by Dove Medical Press Limited (Creative Commons Attribution—Non-Commercial (unimported, v3.0), open-source publishing.

Furthermore, Shen et al. [107] divided the fabrication strategies for nanomedicines with a high drug-loading capacity into four main classes: nanomedicines constructed with inert material carriers (such as MSNs and metal organic frameworks (MOFs) due to their porous structures), those fabricated with drugs as part of the carrier (e.g., polymeric conjugates), nanomedicines that are carrier-free (including drug nanocrystals and amphiphilic drugs), and nanomedicines constructed with different complex strategies (including aqueous noncovalent assembly) (Figure 8).

Most nanomedicines are characterized by a low drug-loading percentage (<10%), and the clinical translation for nanomedicines with such a low loading remains a challenge [106]. For this reason, nanomedicines with a drug loading >10% have attracted much interest [107]. Recently, Matos et al. [108] reviewed the preparation techniques and quantification techniques for EO-based nanosystems.

The loading strategies for EOs can be classified into two major categories related to the fabrication methods and nature of the nanocarriers. The most common strategy is to use an emulsion of the oil/water phase, which can be considered a direct synthesis. The second strategy relies on a post-synthesis method, in which nanoparticles are prepared and the EOs then loaded into the nanoparticles. This strategy applies to inorganic nanoparticles such as MSNs. The loading capacity of EOs in nanoparticulate systems varies depending on the different parameters, including EO structure, nanocarrier, preparation conditions, and method. In Table 1, we list some examples for the loading capacity with both strategies. 

## 3. Controlled Release of EOs from Loaded Nanostructures

The findings support the efficiency of nanosystems in comparison with free EOs. However, many trials are still needed that evaluate EO nanoformulations as anticancer agents, using feasible designs. 

The release of EOs from different nanocarriers (polymeric, molecular complex, inorganic, and lipid) can be affected by their properties (i.e., chemical, mechanical, and stabilizing). The nanoencapsulation of EOs permits the enhancement of a controlled release and cellular uptake, along with the ability to target a specific site [116]. Nanoencapsulation is defined as the encapsulating of flavors as EOs in capsules of sizes ranging from 10 to 1000 nm; this can protect and significantly enhance their medicinal properties, such as bactericidal, virucidal, fungicidal, antiparasitical, analgesic, anticancer, antioxidant, anti-inflammatory, and others [116]. The release of EOs from various nanostructures: nanocapsules, nanoparticles, liposomes, nanoemulsions, solid lipid nanoparticles, and molecular complexes varies and can depend on the various processes involved, such as diffusion, dissolution, desorption, degradation, or different combinations of them [117,118,119,120]. 

In Table 2, we show selected in vitro release studies and analysis techniques, presenting the methods performed for various EOs nanoformulations. 

### 3.1. Controlled Release of EOs from Polymer Nanocarriers

**Figure 9:**
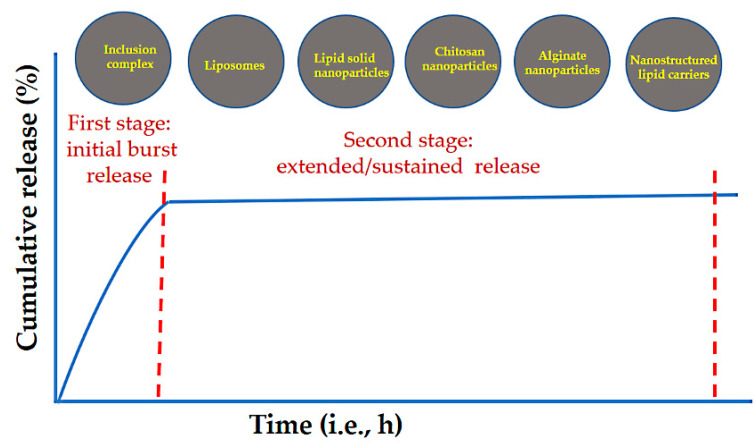
Schematic representation of the two-stage release pattern for EOs from various nanostructures, such as polymers, lipids, and inclusion complexes. As indicated in the literature, this release profile for EOs is the most common.

In this section, we discuss EOs released from polymeric nanocarriers. Polymeric nanostructures have been widely investigated for EO encapsulation. The resulting formulations range in particle sizes from the nanometer to micrometer scale. Biocompatible polymers of natural and synthetic origin are in use [116]. The natural polymers can be classified into two major classes: polysaccharides (e.g., chitosan, alginate, pectin, cellulose, Arabic gum, carrageenan, and zein) and proteins (e.g., albumin, gelatin, soy proteins, and casein). The synthetic polymers include polylactic acid, polyglycolic acid, polylactic glycolic acid, polyvinyl alcohol, and others. As evidenced by a large number of studies, the most common release pattern obtained for EOs from these systems is a two-stage process. In the first stage, an initial burst release occurs, followed by a sustained release as the second stage (Figure 9).

Presently, chitosan alone or formulated with other polymers has been successfully developed for producing different forms of encapsulation (e.g., nanoparticles and nanocapsules). Chitosan is generally recognized as safe (GRAS) by the FDA, reflecting its biocompatibility and increased applications in drug delivery systems. Esmaeili and Asgari [128] studied the release of *Carum copticum* EO from chitosan nanoparticles (30–80 nm). The authors found that the in vitro release profiles indicated two stages: the initial burst for a few hours (within 20 h) and then a sustained release for up to 100 h. Of note, they reported that the release profiles differed with the pH, with a higher release at both stages under acidic and neutral pH conditions. Shetta and co-workers also described the two-stage release pattern from chitosan nanoparticles [113]. They loaded peppermint and green tea EOs into their nanoparticles, yielding a nanosystem with an average size of 20–60 nm. Their in vitro findings revealed a two-stage pattern of release from phosphate-buffered saline (PBS) and acetate buffers under different pH conditions. In the first stage, the initial burst was observed up to 12 h, but in the second stage, the slow release lasted up to 72 h. Their results also indicated a higher release rate under an acetate buffer compared with PBS, with a maximum release of about 75%. The authors further analyzed the release kinetics and found that a release from both buffers followed a Fickian model. In another example, Hasheminejad and co-workers (improving the antifungal activity of clove essential oil encapsulated by chitosan nanoparticles) reported on clove EO encapsulated by chitosan nanoparticles (40 and 100 nm). Under acidic pHs (3 and 5), this release was also two stages, an initial burst of up to 10 days, followed by a sustained release of up to 56 days. These authors further concluded that the amount released changed with the pH. Hosseini et al. [120] used spherical-shaped chitosan encapsulation of oregano EO nanoparticles (40–80 nm) and also identified the two-stage release effect. Using carvacrol, a main component derived from various EOs of thyme, savory, and oregano, Keawchaoon and Yoksan [111] prepared spherical chitosan-loaded carvacrol nanoparticles with sizes of 40–80 nm. Their in vitro findings covering 30 days indicated variable release rates based on the pH, with 53% in an acidic medium, 33% in an alkaline medium, and 23% in a neutral medium after 30 days. This system showed a sustained slow-release rate for carvacrol from these nanoparticles, characterized by Fickian diffusion kinetics. 

On the other hand, Karam et al. [121] described the ability of spherical chitosan nanoparticles (800 nm) to release chamomile EO in one stage for up to 3 days, with a potentially linear pattern based on pHs of 5.5 and 7.4. 

Another strategy is to fabricate a complementary system consisting of chitosan with other polymeric materials [129]. As an example, Hasani et al. [125] fabricated nanocapsules of chitosan/modified starch with lemon EO, yielding a particle size ranging from 340 to 555 nm. Their results demonstrated a prolonged release of lemon EO from prepared nanocapsules with two-stage profiles over 120 h, and the release amount depended on the chitosan:starch ratio. Abreu et al. [130] fabricated chitosan/cashew gum nanocapsules (with an average size of 335–558 nm) consisting of *Lippia sidoides* EO. Their release investigation showed the same pattern of a slow and sustained release. 

The alginate biopolymer, produced by marine algae, is an appropriate matrix for controlling release because of its biocompatibility, biodegradability, safety, low cost, and other properties [131,132,133]. De Oliveira et al. [134] prepared alginate/cashew gum nanoparticles encapsulating *Lippia sidoides* EO, with a size range of 223–399 nm, and evaluated them in in vitro release studies. The results showed a nanoparticle release of about 45–95% of the EO during 30–50 h, following the Korsmeyer–Peppas kinetics model. A group using the same strategy investigated turmeric and lemongrass EOs loaded in chitosan/alginate nanocapsules (about 255 and 230 nm, respectively) for their release performances in PBS medium containing 20% ethanol at pHs 1.5 and 7.4 [122]. The system modulated the release depending on the EO and pH. Under neutral conditions (pH 7.4), the system released approximately 90% of turmeric EO but only 42% of lemongrass EO over 48 h. Under acidic conditions (pH 1.5), the amount of loaded EOs released declined to <70% in the case of turmeric EO and <38% for lemongrass EO over 48 h, with a sustained release pattern.

Regarding other polymeric nanocarriers, we highlight some designs that have been studied for the controlled release of various Eos. Poly (lactic) glycolic acid (PLGA) is an FDA-approved synthetic polymer [135] that is widely used for controlled release. It is considered a biocompatible and biodegradable carrier matrix for drugs. Several studies have shown a two-stage release pattern with PLGA similar to the pattern with chitosan. Giulio et al. [136] used nanocapsules composed of carvacrol at a size of approximately 209.8 nm. Carvacrol release occurred in a two-stage pattern, with a rapid release in the initial phase followed by a slower release for most of the oil, caused by a concentration gradient effect. The two-stage release pattern was also confirmed for eugenol and for the trans-cinnamaldehyde component release PLGA nanocapsules [137]. Another system composed of pectin/chitosan nanoparticles loaded with jasmine EO was investigated for the release kinetics under different pH conditions (pH: 3.0, 5.5, and 7.4) for 48 h [49]. As in most of the other cases, the EO release changed with the pH and was more rapid at pH 3.0 compared with pHs 5.5 and 7.4. The release profiles again showed a two-stage pattern, and the percentage of the release varied with the pH, as follows: pH 3 > pH 7 > pH 5.5. Additionally, these authors reported that the release followed the Korsmeyer–Peppas model, suggesting the diffusion of EO from the nanoformulation matrix. 

Another polymer matrix consists of zein proteins produced from maize, which are FDA-approved as GRAS. Shinde et al. [112] reported the two-stage release of the carvacrol component encapsulated in zein nanoparticles (250 nm). The particles released approximately 80% of the total encapsulated amount for 24 h. A thymol component also showed a two-stage release profile from the zein nanoparticles (stabilized by sodium caseinate–chitosan hydrochloride), with an average size of 200 nm [138]. 

Human serum albumin is another protein reported to deliver EOs and is characterized by its biocompatibility, biodegradability, lack of toxicity, and solubility in water, among other features. Maryam et al. [139] examined carvacrol-loaded human serum albumin nanoparticles into two diameters of ~130 and ~230 nm. With their system, they demonstrated that approximately 21.5% of the total encapsulated carvacrol was released within 3 h (nanoparticles prepared by a desolvation method), whereas about 27% was released within 3 h (nanoparticles prepared by an emulsion/desolvation method). In addition, the system extended the carvacrol releases to 10 days to achieve an ~80% release of the total encapsulated amount, suggesting a long-term release pattern. 

### 3.2. Controlled Release of EOs from Lipids

The lipid nanocarriers can be classified based on materials such as liposomes, niosomes, micelles, nanoemulsions, solid lipid nanoparticles (SLNs), and nanostructured lipid carriers (NLCs) [116]. Liposomes and niosomes are colloidal systems, whereas SLNs and NLCs are solid systems. With a long history dating back to 1970, liposomes are quite commonly used in drug delivery systems [140], and their applications in controlled EOs have been extensively investigated. Generally, liposomes are spherical vesicles distinguished by an aqueous core and an amphiphilic lipid bilayer [141]. They can be classified by size as multilamellar vesicles (>0.5 mm), small unilamellar vesicles (20–100 nm), or large unilamellar vesicles (>100 nm) [142,143]. Of interest, liposomes are extremely biodegradable, biocompatible, and nontoxic delivery vehicles [144] and, thus, are well-known for their use in incorporating hydrophobic, hydrophilic, and amphiphilic agents. To improve the biopharmaceutical properties of EOs, Risaliti et al. [145] loaded Greek sage and rosemary EOs in liposomes with an average particle size of ~200 nm. In vitro release experiments showed a linear kinetic release for the EOs of about 40% within 1 h and 100% within 3 h. Consequently, the obtained release pattern is one with a short-term profile. In contrast, a longer sustained release pattern was obtained for an *Artemisia annua* L. (sweet wormwood and sweet sagewort) EO [146]. The prepared nanoliposomes containing this EO prolonged the release over 14 h (100% released). 

Due to the short-term release effect with traditional liposomes, an alternative way to extend the release is being developed, using an EO in CD in a liposome formulation. Along these lines, the inclusion of EO components in hydroxypropyl-β-CD-in-lipoid S100/cholesterol liposomes is a promising approach reported by Hammoud et al. [147]. The tested EO components were monoterpenes (eucalyptol, pulegone, terpineol, and thymol) and phenylpropenes (estragole and isoeugenol). These authors concluded that the system could extend the release and activity of the EO components. In addition, the EO encapsulation content considerably affected the release profiles with this system. Going further, the research team also showed that the EO release rate from this liposome formulation varied with the loading degree, particle size, and location of the EO components in the liposome [148]. In another colloidal system relying on micelles, Thonggoom et al. [149] developed a micellar formulation for a clove EO and found that the system was suitable for sustained EO release.

Solid lipid formulations with SLNs and NLCs have gained much interest in controlling the EO release. In a traditional system, Zhao et al. [150] formulated SLNs composed of Yuxingcao EO with sizes between 171 and 812 nm. Their results indicated that the nanoparticles released EO in a sustained profile for up to 48 h. Another example, reported by Rodenak-Kladniew et al., used linalool, a major component in many EOs [151]. These authors loaded linalool into SLNs as a potent formulation for cancer. The obtained nanoparticles were spherical, with particle size diameters from 90 to 130 nm and a controlled linalool release over 72 h. In another study, Moghimipour et al. [152] prepared spherical SLNs composed of a *Z. multiflora* EO with a mean particle size of 650 nm. They found that ~93.2% of the EO content was released after 24 h, suggesting a quick initial release. 

In recent years, NLCs have been developed for EOs [153]. NLCs are characterized by their unique lipid properties as a mixture of solid and liquid lipids compared with traditional lipid materials such as liposomes and solid nanoparticles. For these reasons, NLC nanostructures are being considered for the sustained release of EOs. Vieira et al. [154] loaded sucupira oil into the NLCs, yielding loaded nanoparticles from ~150 nm to 160 nm. They found a release of the EO over 8 h, fitting it to the first-order kinetics. Consistent with the importance of NLCs for releasing EOs are the recent reports of solid inclusion complexes maintaining a sustained release of a *Lippia origanoides* EO. The formulation was fabricated using NLCs encapsulated with the hydroxypropyl-β-CD inclusion complexes containing EOs [115]. The results indicate that the system controlled EO release in zero-order kinetics (~20% released after 12 h) compared with CD alone, which fit to Hixson–Crowell kinetics (~50% released after 3 h). As evidenced from a study by Manzar et al. [114], applying the NLCs (<150 nm) led to a sustained liberation effect for *Cuminum cyminum* EO from these nanoparticles in the gastrointestinal tract. 

### 3.3. Controlled Release of EOs from Inclusion Complexes

Inclusion complexes are of increasing interest for EO nanoformulations for a wide range of therapeutics. CDs are among the most applied inclusion complexes for EO encapsulation [155,156,157]. Their main feature is seven glucopyranose units with an internal hydrophobic/lipophilic cavity of 0.6 nm (diameter) that facilitates the formation of inclusion complexes with aromatic moieties, i.e., EOs [158]. Matshetshe et al. [118] studied the release of cinnamon EO from the spherical β-CD/chitosan nanoparticles with particle sizes of 100–410 nm. The results of their in vitro cumulative release experiments revealed a biphasic release effect, with an initial burst release pattern (~68% within 12 h) and then an extended–release pattern (from 12 to 120 h). This pattern reflects the Fickian diffusion mechanism. Similarly, spherical hydroxypropyl beta-CD nanoparticles with an average particle size of ~166 nm display a two-stage release pattern for clove EO [159]. Furthermore, the myrcene component encapsulated in CD matrices (CD, β-CD, γ-CD, and 2-hydroxypropyl-β- (HP-β-CD)) shows first-order kinetics regarding the diffusion mode [160].

### 3.4. Controlled Release of EOs from Inorganic Nanocarriers

Although many inorganic nanostructures have attracted interest in the field of drug delivery systems for a wide range of therapeutic agents and diseases, their contributions as delivery systems for EOs are still quite limited. Different inorganic nanomaterials are available, such as mesoporous silica nanoparticles (MSNs), zinc oxide nanoparticles, and magnetic nanoparticles. Due to a lack of data regarding the use of inorganic nanoparticles for EO encapsulation, we discuss only MSNs, which have shown some promise in various applications. Indeed, even for MSNs, few studies are available, but we anticipate that the volume will soon increase. MSNs occur in many forms (e.g., MCM-41, SBA-15, KCC-1, and KIT) and have been extensively used as drug delivery nanocarriers because of exceptional properties. They are characterized by stability (chemical and mechanical), easy synthesis and functionalization, a large surface area, tunable pore sizes and volumes, good biocompatibility, controlled drug release under different conditions, and a high drug-loading capacity, enabling multifunctional purposes and targeting [161,162,163,164,165,166,167,168,169,170,171]. 

The evidence from in vivo preclinical evaluations supports a good safety profile for MSNs. Compared with organic delivery systems used for the controlled release of drugs (e.g., lipid nanoparticles, inclusion complexes, and polymeric nanoparticles) [172,173], which show fast degradation, corrosion, and initial diffusion, MSNs can support long-term releases. In a recent study, SBA-15 proved able to load about 70.8 wt.% of the thymol component [110] and showed a long-term release pattern. These authors found that only 27% of the total loaded thymol was released within the first 24 h, but the release continued for another 34 days (to ~70% of the total amount of thymol). Another example of using MSNs for long-term EO release was reported by Cadena et al. [13]. They found that the release of a grafted cinnamaldehyde component (the main component in cinnamon EO) from MSNs varied with the presence or absence of lactose capping onto the loaded MSN surfaces. The results showed that the total cinnamaldehyde grafted to MSNs was released after 24 h, with an initial burst release of approximately 50% in the first hour. In contrast, no release for cinnamaldehyde grafted to lactose-capped MSNs was observed even after 48 h, indicating the role of surface capping in longer-lasting EO component releases from MSNs. 

## 4. Targeted Nanodelivery Systems for Anticancer Applications

**Figure 10:**
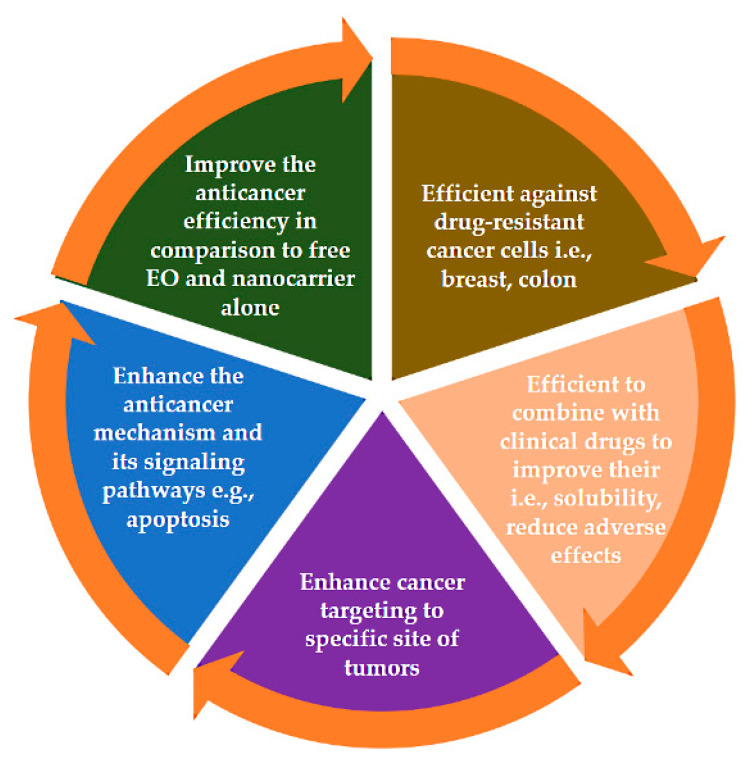
Schematic illustration of the impacts of nanoformulations with EOs as compared with free EOs in cancer applications.

**Figure 11:**
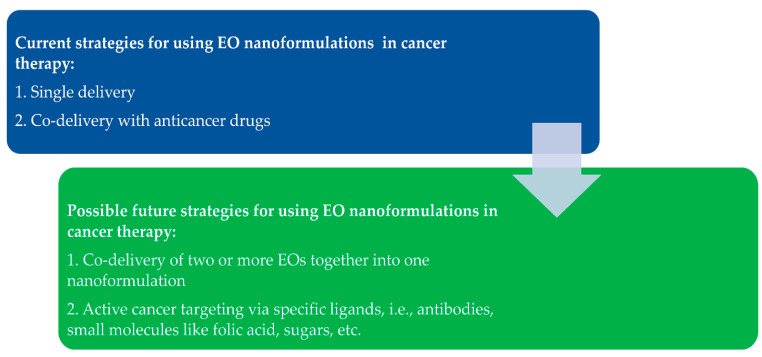
Schematic representation of the current and potential future research strategies for nanoformulation delivery systems using EOs.

Drug delivery systems based on nanomedicine are tailored to achieve specific site-targeting cancer cells rather than normal cells. In the last few years, researchers have sought to tailor various promising delivery designs with EOs established for cancers. Here, we highlight several systems that show efficient anticancer effects compared with free EOs. The improvements include enhanced anticancer effects, improved anticancer mechanisms of action, and reduced side effects of anticancer drugs in cases involving combination treatments (Figure 10). As indicated by the literature described in Figure 11, the research reflects possible future strategies for investigating nanoformulation delivery systems based on EOs. 

### 4.1. Nanodelivery Systems for Breast Cancer 

Very recently, Rana et al. [174] demonstrated that *Juniperus squamata* EO-loaded functionalized nanographene oxide exhibits higher cytotoxic effects against MDA-MD-231 human breast cancer cells compared to free EOs. In this regard, the proliferation of human breast cancer cells were found to be inhibited when cells treated *Heracleum persicum* EO nanoemulsion associated with a high antioxidant potential [175]. By the way, a codelivery made of farnesol–gingerol by niosomal formulation results in enhanced anticancer activity when tested on breast cancer cells [176]. Salehi et al. [177] showed that, compared with free EOs, a nanoemulsion consisting of *Zataria multiflora* EO enhanced the selective anticancer activity against invasive MDA-MB-231 breast cancer cells while showing minimal toxic effects on normal fibroblast cells (L929). Their findings suggested that the system induces apoptosis by generating ROS, triggering mitochondrial membrane permeabilization and damaging the DNA. 

Similarly, Periasamy et al. [178] reported that a stable *Nigella sativa* L. EO (black seed) nanoemulsion exerted a potent antiproliferative effect on MCF-7 cancer cells by modulating their nucleocytoplasmic morphological features (including cell membrane blebbing, cytoplasmic vacuolation, chromatin marginalization, and nucleus fragmentation) and inducing apoptosis. Al-Otaibi et al. [48] prepared a codelivery system consisting of the antineoplastic agent mitomycin C with ginger EO or frankincense EO in a nanoemulsion formulation and assessed the anticancer effects against HeLa cervical cancer cells and MCF-7 breast cancer cells. Of these treatments, the nanoformulation with ginger EO had the strongest apoptotic effect on MCF-7 cells, suggesting the potential for a codelivery nanoformulation with EOs to enhance the solubility and efficiency of the clinical drugs. In search of new alternative agents in the face of drug resistance, Salehi et al. [105] developed a system of citrus–pectin nanoemulsion-encapsulated *Zataria multiflora* EOs. The nanoformulation progressively improved the killing of drug-resistant cell lines (MCF-7 and MDA-MB-231 breast cancer cells) and spheroids. These authors reported that the system activated apoptosis via different signaling pathways, including ROS (increase), mitochondrial membrane potential (loss), DNA (damage), and G2- and S-phase arrest in MDA-MB-231 cells and spheroids. Attallah et al. [49] recently reported that the nanoparticulate system of jasmine EO/pectin/chitosan nanoparticles exerted potent anticancer effects against MCF-7 cancer cells compared with pure jasmine EO, yielding an approximately 13-fold improvement while producing no toxicity against L-929 normal cells. The normal cells were, in fact, rather enhanced with a nanosystem treatment, suggesting that the nanoformulation might even have increased their viability. Another confirmation of the nanoformulation potential against breast cancer was reported for *Cyperus articulatus* EO-loaded nanoparticles, which highly inhibited MDA-MB-231 breast cancer cells after incubation for 48 h [179]. The use of EO through codeliveries with drugs is a new way to eliminate adverse side effects, as Alkhatib et al. [180] examined in terms of the neurotoxicity and nephrotoxicity associated with ifosfamide. They obtained the greatest apoptotic action against MCF-7 breast cancer cells with the drug incorporated into lemon or salvia EO-based nanoemulsion, suggesting an enhanced solubilizing of the drug with this formulation. Recently, Panyajai et al. [181] developed nanoformulations that significantly enhance the anticancer effects, especially against MCF-7, and show a superior internalization into cancer cells. 

### 4.2. Nanodelivery Systems for Lung Cancer

A few studies have examined the effects of nanosystems against lung cancer cells. Khan et al. [182] tested a carvacrol nanoemulsion component on A549 adenocarcinoma lung cells. Their in vitro results demonstrated that the system kills cancer cells by apoptosis mediated through ROS production, p-JNK, Bax, Bcl2, cytochrome c, caspase activation, and mitochondrial suppression. During in vivo studies through the oral application of athymic nude mice, the model showed a strong antitumor potential, signifying the importance of this system as a promising candidate for lung cancer therapy. Furthermore, the use of chitosan nanoparticles loaded with *Morinda citrifolia* EO was found to enhance the antitumor activity (via morphological modification, nuclear damage, ROS generation, and cell cycle arrest) against A549 human lung cancer cells while exerting a minimum cytotoxicity against human red blood cells [183]. 

Another system consisting of black seed EO coated in gold nanoparticles proved to be highly effective at inhibiting A549 lung cancer cells compared with Au nanoparticles or the EO alone [184].

### 4.3. Nanodelivery Systems for Liver Cancer 

Nanocapsules containing an *Origanum glandulosum* Desf. (oregano) EO showed a stronger cytotoxic inhibitory effect on the HepG2 liver cancer cell line (IC_50_ of 54.93 μg/mL) than oregano EO alone, with an IC_50_ of 73.13 μg/mL [101]. A delivery system using niosomes composed of *Trachyspermum copticum* EO resulted in a higher cell toxicity against HepG2 cancer cells in comparison with EO alone, suggesting the anticancer potential of the nanosystem [185]. 

### 4.4. Nanodelivery Systems for Colon Cancer

Despite the attempts to find novel therapies for colon cancers, few studies have been published. Recently, the encapsulation of nerolidol (naturally found in EOs like citronella) into solid lipid nanoparticles has enhanced the anticancer activity against human colorectal cells [186]. Khatamian et al. [187] prepared carvi oil nanoemulsions and investigated their anticancer activity on HT-29 human colon cancer cells and their apoptotic properties. These authors found that the nanoformulations significantly inhibited the viability of HT-29 cancer cells (IC_50_: 12.5 µg/mL) compared with normal human umbilical vein endothelial cells (HUVECs) (IC_50_: 12.5 µg/mL). In addition, nanoemulsions significantly upregulated caspase-3, indicating a potent apoptosis induction in the colon cancer cells. Another system was developed based on bergamot EO nanoemulsions and showed a measurable cytotoxic activity against Caco-2 colon cancer cells compared with free EOs, indicating an enhanced anticancer effect [188].

### 4.5. Nanodelivery Systems Fabricated for Brain Cancer

Detoni et al. [189] evaluated the *Zanthoxylum tingoassuiba* EO loaded into liposomes against glioblastoma cells. Their results showed a significant impact of the liposomes containing EO on apoptosis induction, suggesting a potential role in the inhibition of glioblastoma and a promising alternative approach. Confirmation of the increased activity against SH-SY5Y human neuroblastoma cells was reported by Celia et al. [190] for bergamot EO formulated into liposomes. Likewise, an in vitro study of U-138MG human glioblastoma cells indicated that nanoemulsions containing a *Drimys brasiliensis* EO reduced the cancer cell numbers, showing a potent effect of the nanoemulsion [191]. 

**Figure 12:**
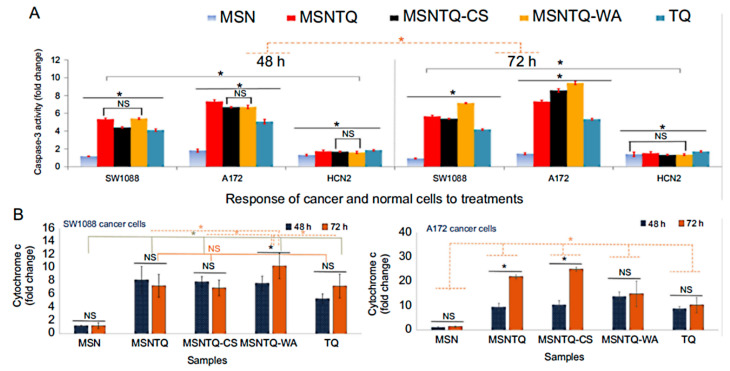
The molecular mechanisms of targets of core nanoformulation (MSNTQ), core–shell nanoformulations (MSNTQ-CS and MSNTQ-WA), and thymoquinone (TQ) in a free form on brain cancer cells (SW1088 and A172) and normal brain cells (HCN2) after 48 and 72 h of incubation. (**A**) Caspase-3 activation in the fold change measured by ELISA for all treatments, and (**B**) cytochrome c intracellular release in the fold change measured by RT-PCR for all treatments. All data are expressed as the mean ± standard deviation. The differences are expressed with * at *p* < 0.05 based on the least significant difference. Nonsignificant differences are marked as NS. In the case of caspase-3, the solid gray line indicates the significance between cell lines, and the dashed orange line indicates differences between incubation times. Reproduced from Ref. [99], Dove Medical Press Limited, an open-access source.

In a study of the nanoformulation of an eugenol EO component loaded into chitosan nanoparticles, the system exhibited potent effects against rat C6 glioma cells in vitro, persuasively inducing apoptosis, along with inhibiting the metastasis of these cells [192]. Recently, we developed a core–shell nanoformulation system for thymoquinone, the most abundant component of *Nigella sativa* seeds EO [99]. The system consisted of mesoporous silica spheres loaded with thymoquinone (as a core), then coated with whey protein–Arabic gum or a chitosan–stearic acid complex (as a shell coating). The results indicated that the system significantly killed glioma cancer cells through apoptosis-mediated pathways, including caspase-3 activation and cytochrome c triggers (Figure 12). These findings support the potential of the thymoquinone-based nanoformulation as a candidate treatment for brain cancer. Very recently, in an in vivo study, Zhang et al. [193] tested a nanoemulsion system consisting of *Pinus koraiensis* EO. The system effectively inhibited the growth of the tumors in MGC-803 tumor-bearing nude mice (via intragastric administration) by promoting apoptosis. Furthermore, immunohistochemical examinations revealed an important role for nanoemulsion in the downregulation of the YAP1/TEAD pathway and its target proteins (CTGF, AREG, and GLI2), regulating the HIPPO/YAP and associated signaling pathways. The authors concluded that the systems could provide a theoretical basis for deep practical applications of EOs.

### 4.6. Nanodelivery Systems for Other Cancers

Killing cancers by combining EOs and drugs has attracted considerable interest. The effects on HeLa cells of a formulation system consisting of bleomycin with a cinnamon EO nanoemulsion were recently reported [194]. These authors concluded that, compared with free drugs, the nanoformulation enabled the more efficient killing of cancer cells, suggesting a greater impact of combination EOs with drugs as a new route for combating cancers. For another type of cancer, oral squamous cell carcinoma, the therapeutic potential of a celery seed EO nanoformulation using nanoemulsion was assessed [195]. The authors found that the nanoformulation was extremely efficient at inhibiting cell proliferation by reducing anchorage-independent cell growth, disrupting colony formation, and inducing apoptosis. 

Studies have also suggested promising effects against several other cancers. One using a nanoemulsion with spearmint EO showed effects against oral carcinoma [196]. The treatment of tongue carcinoma cells with a cumin EO nanoemulsion diminished the colony formation [197]. Solid lipid nanoparticles with a *Zataria multiflora* EO have shown an anticancer efficacy against A-375 melanoma cells [198]. Another system consisting of a chitosan nanocarrier encapsulating a black seed EO revealed enhanced antiproliferative properties against PC3 prostatic cancer cells [199]. With gold nanoparticles encapsulating black seed EO, there was an improved anticancer effect reported against A549 lung cancer compared with EO alone [184]. Nanoemulsions fabricated with ginger EO and frankincense have been evaluated against various cancers and have shown promising anticancer activity against HeLa cells [48]. Han et al. [200] developed linalool-incorporated nanoparticles for epithelial ovarian cancer, and the system investigated in vitro and in vivo studies (mice were administered via the i.p. route); they found that this delivery is effective and promising against ovarian cancer. 

## 5. Conclusions and Future Perspectives

Essential oils (EOs) are promising therapeutic natural agents comprising hundreds of chemical compounds with untapped medical potential. They are distinguished by complex chemical structures not present in other natural agents and have the advantages of promising medical properties, abundant availability, cost-effective production, and potential for large-scale production. However, there are major limitations on their clinical use, i.e., low stability, high volatility, low solubility, poor bioavailability, and insufficient specific targeting. Nanomedicine offers strategies to overcome these limitations by the application of nanotechnology. The nanotechnology approach includes the loading of nanostructures with EOs or their encapsulation, improving the release profiles and improving the targeting of selected cells. 

The nanostructures in question are: nano- and microcapsules, nanoparticles conjugated with EOs, nanogels, EO-loaded nanoparticles, and nanoemulsions, which are the most investigated forms of nanoformulations. Several preclinical tests, including cell tests and in vivo tests, demonstrated an improved control of the EO release compared with the application of free EOs. It is also possible to achieve a two-stage release pattern. Further, it is possible to improve the targeting of cancer cells with EOs.

Nanoformulations also improve the EO anticancer efficiency against drug-resistant cancer cells, such as breast cancer cell lines, with EOs loaded alone or combined with clinical drugs. Furthermore, an emerging research direction is the combination of EO-based nanostructures with anticancer drugs, which may improve the solubility, bioavailability, and activity of the anticancer therapies. 

For future research directions for cancer therapy and to improve cancer targeting by EO-based nanoformulations, we suggest exploring their conjugation with ligands such as antibodies or folic acid. Another approach to investigate is the use of inorganic nanostructures such as mesoporous silica nanospheres, hydroxyapatite nanoparticles, and zinc oxide nanoparticles to extend a controlled release. Additionally, codelivery systems that load two or more EOs or combine an EO with an anticancer drug should be evaluated in animal models. Since there is a huge number of EOs, studies of more EOs or their free components in nanoformulation nanosystems should be conducted to offer many choices for anticancer therapy. 

In our opinion, the application of nanomedicine for EOs will expand their pharmaceutical and biomedical applications over traditional use and add value to cancer treatments. The path is open to developing EO-based nanoformulations for further translation into clinical use against cancer.

## Figures and Tables

**Table 1:** Loading capacity and method for various EOs with different nanocarriers.

EO	Nanocarrier	Loading Capacity	Loading Method	References
Jasmine	Pectin/chitosan nanoparticles	0.43–6.06%	Direct preparation method	[49]
Zedoary	Chitosan-grafted mesoporous silica nanoparticles	41%	Gate-penetration by supercritical CO_2_	[109]
Thymol compound	SBA-15 mesoporous silica nanoparticles	~71	Post-fabrication method	[110]
Carvacrol compound	Chitosan	Up to 21	Direct preparation method	[111]
Carvacrol compound	Zein	Up to 13%	Direct preparation method	[112]
Peppermint and green tea	Chitosan nanoparticles	Up to 23%	Direct preparation method	[113]
*Cuminum cyminum*	Nano lipid carrier	Over 70%	Direct preparation method	[114]
*Lippia origanoides*	Nanostructured lipid carriers/cyclodextrin inclusion complexes	2.6%	Direct preparation method	[115]

**Table 2:** Experimental in vitro release and analysis techniques of EOs from nanoformulations of different nanocarriers.

EO	Nanocarrier	In Vitro Experiment	Analysis Technique	References
Peppermint and green tea	Chitosan nanoparticles	Lyophilized encapsulated nanoparticles were placed in a dialysis bag containing release media: PBS or acetate buffer. This bag was incubated in an additional volume of the release medium. Left under gentle shaking at room temperature. Sampling: a predetermined volume of release media was taken at specific intervals, then an equal volume of fresh release media was added during the entire experiment.	UV–Vis spectrophotometer	[113]
Chamomile	Chitosan nanocapsules	Freeze-dried nanocapsules placed in PBS buffer release medium containing 0.5% Tween 80 adjusted to pH 5.5 or 7.4. The solution was orbitally stirred (150 rpm) at 37 °C. Sampling: at predetermined times, the solution was washed twice with hexane (using a separatory funnel), and the supernatant was used for analysis.	Gas chromatography (GC)	[121]
Turmeric and lemongrass	Chitosan-alginate nanocapsules	The loaded nanocapsules were dispersed in a small volume of PBS solution and put in a dialysis bag, then surrounded by PBS containing 20% ethanol adjusted to pH 1.5 and 7.4. Next, the solution was incubated at 37 °C under gentle agitation. Sampling: a specific volume was withdrawn and replaced with fresh medium.	UV–Vis spectrophotometer	[122]
Lavender	Hydrogel microparticles	Headspace GC	GC analysis	[123]
Pomelo citrus	Zein-alginate solid nanoparticles	The lyophilized encapsulated nanoparticles were placed in simulated fluid into dialysis bags, sealed, and suspended in the same simulated fluid. The solution was stirred (100 rpm) at 37 °C. Sampling: a known volume was taken from the solution and replaced with the same volume of fresh simulated fluid. For analysis, and the withdrawn sample was partitioned with an equal volume of ethyl acetate.	UV–Vis spectrophotometer	[124]
Lemon	Chitosan-modified starch nanocapsules	Freeze-dried nanocapsules were placed in a microtube containing 40% ethanol. This mixture solution was incubated at room temperature under stirring. Sampling: at specified time intervals, the samples were centrifuged. The supernatant was removed for analysis.	UV–Vis spectrophotometer	[125]
Cinnamon	Poly(e-caprolactone) electrospun nanofibers	The nanofibers were placed in a simulated dissolution medium at 37 °C, and the paddle was set at 100 r/min. Sampling: at fixed time intervals, aliquots were taken and replaced with an equal volume of fresh release medium. The samples were filtered and diluted to be used for analysis.	UV–vis spectrophotometer	[126]
Eucalyptus	Β-cyclodextrins, functionalized zeolite materials	Headspace extraction with GC	GC analysis	[127]
Cinnamon	Β-cyclodextrin/ chitosan nanoparticles	The loaded nanoparticles were dispersed in acetate and phosphate buffers and vortexed at room temperature. Sampling: at predetermined specific time intervals, the samples were centrifuged (for 5 min at 14,000 rpm); then, aliquots of the supernatant were withdrawn (for analysis) and replaced with the same volume of buffer, and the experiment continued.	UV–Vis spectrophotometer	[118]

## Data Availability

Data is contained within the article.
